# New Frontiers for Applications of Thermal Infrared Imaging Devices: Computational Psychopshysiology in the Neurosciences

**DOI:** 10.3390/s17051042

**Published:** 2017-05-05

**Authors:** Daniela Cardone, Arcangelo Merla

**Affiliations:** Infrared Imaging Lab, ITAB Institute for Advanced Biomedical Technologies, Department of Neuroscience, Imaging and Clinical Sciences, University of Chieti-Pescara, Chieti 66100, Italy; arcangelo.merla@unich.it

**Keywords:** thermal imaging systems, computational psychophysiology, autonomic nervous system, thermal detectors, microbolometers

## Abstract

Thermal infrared imaging has been proposed, and is now used, as a tool for the non-contact and non-invasive computational assessment of human autonomic nervous activity and psychophysiological states. Thanks to a new generation of high sensitivity infrared thermal detectors and the development of computational models of the autonomic control of the facial cutaneous temperature, several autonomic variables can be computed through thermal infrared imaging, including localized blood perfusion rate, cardiac pulse rate, breath rate, sudomotor and stress responses. In fact, all of these parameters impact on the control of the cutaneous temperature. The physiological information obtained through this approach, could then be used to infer about a variety of psychophysiological or emotional states, as proved by the increasing number of psychophysiology or neurosciences studies that use thermal infrared imaging. This paper presents a review of the principal achievements of thermal infrared imaging in computational psychophysiology, focusing on the capability of the technique for providing ubiquitous and unwired monitoring of psychophysiological activity and affective states. It also presents a summary on the modern, up-to-date infrared sensors technology.

## 1. Introduction

Understanding psychophysiological and emotional states of a conversational interlocutor is a key point for establishing a proper communication, tying social and affective bonds, choosing social strategies and setting a contingent interaction. Typically, psychophysiological and emotional states have been, and still are, assessed through behavioural analysis and/or the measurements of several Autonomic Nervous System (ANS) parameters, like galvanic skin response [1], hand palm temperature [2], modulations of heart beat and/or breathing rate [3], and peripheral vascular tone [4]. Classical approach for monitoring these ANS variables requires the use of contact sensors or devices, thus resulting invasive for the subject and, overall, biasing the estimation of the emotional state, since the compliant participation of the individual is required. 

To overcome the limitations of contact sensors, computational psychophysiology based on imaging approaches has been suggested. Among these, thermal InfraRed (IR) imaging has been proposed as a potential solution for the quantitative assessment of several psychophysiological parameters associated with ANS activity [5].

Thermal IR imaging or infrared thermography is a widespread imaging technique used to accurately evaluate the thermal distribution of a body without any contact between the sensors and the body itself. Thermal imaging devices, or thermal cameras, are able to capture the infrared radiation emitted by the body and to convert it into a radiometric thermal image that is a digital map of the superficial temperature distribution of the body itself. 

During the last decades, thermal IR imaging has become a cutting edge technique in many application fields, from mechanical and electrical inspection to building diagnostics, from optical gas detection to automation and industrial safety. Up-to-date IR detectors, both based on cooled and uncooled technology, guarantee a high thermal resolution and accuracy. Furthermore, the impressive spread of IR technology, together with the miniaturization of IR detectors, conducted the manufacturer houses to produce even mobile thermal cameras.

In the biomedical field, thermal IR imaging allows the contactless and non-invasive recording of the cutaneous temperature through the measurement of the spontaneous thermal irradiation of the human body. 

Quantitative parameters modelling the cutaneous temperature distribution and time-evolution are often obtained using bioheat transfer models (BHTM) [6,7]. The first BHTM equation was elaborated by Pennes [8]. He developed a blood perfusion model based on experimental measurement of the human forearm. This model allowed the estimation of the heat transport and temperature variation within biological tissue, hypothesizing that the temperatures of blood incoming and outgoing capillaries were both constant for any small volume of tissue. Among the others, a new version of BHTM was recently developed by Shrivastava et al. considering the conservation of thermal energy in a heated, vascularised and finite tissue [6]. The model is constituted by two linear, coupled differential equations depending on tissue volume averaged temperature and blood volume averaged temperature. 

Through the use of IR thermography, it is also possible to obtain information on the ANS activity, since, beyond being the main regulator and controller of some vital functions, such as heart rate, digestion, respiratory rate and perspiration, it directly controls the thermal exchanges between the human body and the surrounding environment. The human face is of special importance since it is naturally exposed to social communication and interaction, thus offering an excellent region for computational psychophysiology based on thermal IR imaging. In fact, several autonomic parameters such as heart rate, cutaneous blood perfusion, breathing rate, and the sudomotor response [9,10,11,12,13,14,15,16,17] have been estimated through the analysis of the modulation of facial cutaneous temperature.

The reason that makes thermal IR imaging particularly suitable for the neurosciences and applied psychophysiology is the possibility to assess the psychophysiological state of one or more subjects at once, while preserving an ecological context of interaction, which is guaranteed by the absence of any contact device. In addition, modern thermal IR imaging devices can rely on both high spatial (up to 1280 × 1024 pixels) and temporal recording resolution (up to 200 full frames per seconds).

Given the proper choice of IR imaging systems, optics, and solutions for tracking the regions of interest (ROIs), it is also possible to avoid any behavioural restriction on the subject [18,19]. This possibility is particularly important, for example, in psychometrics studies or human artificial agent interaction or developmental psychology fields, with non-collaborative subjects like children [20,21]. Although the present review is focused on applications of thermal IR imaging on computational psychopshyiology and neurosciences, it is worth noticing that the impressive advancement of thermal IR imaging is positively impacting on the medical diagnostics. Fields of interest are the vascular disorders, endocrinology, traumatology and orthopaedics, neurology, neonatology, oncology. A very strong interest is regarding also sport medicine [22]. An up to date review of thermal IR imaging for biology and medicine has been recently provided by Vardasca and Gabriel [23]. Areas of interest are microvascular imaging, diagnosis of venous diseases, diagnosis of orthopaedic injuries in childhood, total body cryotherapy, evaluation of myogenous temporomandibular disorders and myofascial trigger points, assessment of physical fitness level, monitoring and prevention of sport injuries, thermal characterization of swimming technique, application to safety studies of vaccines, oncobiology, racehorse performance. The possibilities for using thermal IR imaging in medicine and biology are continuously increasing, with a growing attention to the technology and its development. 

In the same way, detection and recognition of emotional states through thermal IR imaging have gained an increasing interest. An interesting review on this topic was conducted by Clay-Warner and Robinson in 2014 [24]. More recently, Salazar-López et al. investigated the cognitive neuropsychology of emotions during emotional tasks [25], while Latif et al. explored the suitability of IR imaging technique for affect detection by means of thermal image feature extraction using Gray Level Co-occurrence Matrix (GLCM) [26]. Finally, another promising research field concerns face recognition by means of thermal imaging. In the last years, Cho et al. presented a method for face recognition based on the identification of vein bifurcation point pattern and gravity center of the thermal face, by means of Modified Hausdorff Distance [27]. An interesting review on this topic has been conducted by Ghiass et al. [28], through the description of several emerging methodologies in the field and the references of the main available databases of infrared facial images. More recently, Hermosilla et al. presented a novel method for face recognition based on the fusion of thermal and visible features through the use of genetic algorithms [29]. 

This paper reviews the state of the art in the field of thermal IR imaging-based computational psychophysiology, with a special emphasis on the technological aspects of modern thermal cameras.

## 2. Infrared Sensors Technology

IR imaging technology was introduced from the first half of the 20th century [30]. Since its birth, it is possible to recognize three generations of infrared cameras [31,32]: the first generation cameras were characterized by a single element detector, combined with two scanning mirrors to create infrared images. Their main disadvantage was that they suffered from saturation problems. Saturation indicates the limit of the highest irradiance that can be measured by a detector. For digital sensors, since incident photoelectrons are converted in charges, each detector can store a maximum amount of charges known as the *full well capacity* [32].

The second generation cameras were characterized by an increase in the number of detectors, positioned in a large linear array or in two small 2-D array. 

The third generation cameras, i.e., the ones currently used, are characterized by large focal plane array (FPA) detectors, thus increasing the reliability and sensitivity of such infrared systems [33]. The main innovations characterizing modern cameras are the increased number of pixels, the higher thermal sensitivity, and the increased acquisition frequency. Specifically, new materials for detector with improved thermal sensitivity are now used, and high-density focal plane arrays (up to 1280 × 1024 pixels) are currently available on the commercial market. Moreover, read-out circuitry using on-chip signal pre-processing led to the availability of commercial and user-friendly infrared camera systems. The thermal sensitivity has been substantially reduced to less than 30 mK (20 mK for nitrogen cooled cameras) with a spatial resolution of 25–40 μm. 

The description of the factors qualifying the performances of a thermal imaging system is reported in Appendix A.

### 2.1. Modern IR Thermal Detectors

FPA detector technologies are divided into two categories: thermal detectors (uncooled) and quantum detectors (cooled). Modern uncooled detectors use sensors whose working mechanism is based on a change of resistance, voltage or current when heated by IR radiation. Uncooled detectors are mostly composed by pyroelectric and ferroelectric materials or based on microbolometer technology. The thermal signal depends upon the radiant power but not upon its spectral content, i.e., it is wavelength independent [34]. Some of the materials used for these sensor arrays are amorphous silicon (a-Si), lead zirconium titanate (PZT), vanadium oxide (VOx), lead lanthanum zirconate titanate (PLZT), lead scandium tantalate (PST), lead lanthanum titanate (PLT), lead titanate (PT), lead zinc niobate (PZN), lead strontium titanate (PSrT), barium strontium titanate (BST), lanthanum barium manganite (LBMO), barium titanate (BT), SbSi and polyvinylidene difluoride (PVDF). Nowadays, VOx microbolomiter arrays are the most widely used technology for uncooled detectors. One of the most important parameters for uncooled detectors is the thermal conductance of the material (G_th_). An increase in thermal conductance, due to improvements in material processing technique, enhances sensitivity, because Noise-Equivalent Temperature Difference (NEDT) is proportional to $\sqrt{G_{\mathrm{th}}}$, at the expense of time response, which is in turn inversely proportional to G_th_ [35].

Cooled detectors (i.e., quantum detectors) are made from materials such as InSb, InGaAs, PtSi, HgCdTe (MCT), and layered GaAs/AlGaAs for Quantum Well Infrared Photon (QWIP) detectors. Nowadays, HgCdTe is the most widely used semiconductor material for cooled IR detectors. Beyond ensuring the use of large number of pixels, high frame rate, and a high thermal resolution, it is characterized by a multicolour functionality, i.e., multiband detection capabilities [36].

The working process of a quantum detector is based on the change of state of electrons in a crystal structure reacting to incident photons. Incident photons, with sufficient energy, when hitting the detectors material, stimulate the electrons in the valence band, causing their movement in the conduction band. Thus the detector can carry a photocurrent, which is proportional to the intensity of the incident radiation. These detectors are characterized by a selective wavelength dependence of the response per unit incident radiation power. They have both perfect signal-to-noise performance and a very fast response [34]. These detectors are generally more sensitive than thermal detectors. However, they require cooling, sometimes down to cryogenic temperatures using liquid nitrogen or a small Stirling cycle refrigerator unit [37]. 

### 2.2. Theory of Thermographic Measurement

**Figure 1:**
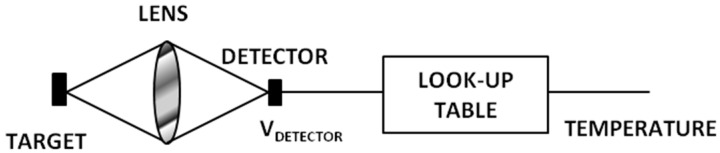
A thermal imaging system whose output is provided in temperature units (Fahrenheit, Celsius, Kelvin, or Rankine) (Adapted from [38]).

Thermal IR cameras convert the IR radiation into an electric output, i.e., a voltage output. The conversion scheme is illustrated in Figure 1 [38]. 

The voltage output comes out from the infrared radiation according to Equation (1):

(1) $$V_{\mathrm{DETECTOR}}=k\cdot R_{D}\left(\lambda \right)\cdot W_{\mathrm{tot}}\left(\lambda ,T\right)$$

where $V_{\mathrm{DETECTOR}}$ is the detector output voltage, k is a constant depending on the specific optics and detectors, $R_{D}\left(\lambda \right)$ is the detector’s responsivity (i.e., output voltage per input radiant power) and $W_{\mathrm{tot}}\left(\lambda ,T\right)$ is the IR emitted energy from the target object. In order to switch from the output voltage to the correct temperature value, a calibration process is needed [38].

**Figure 2:**
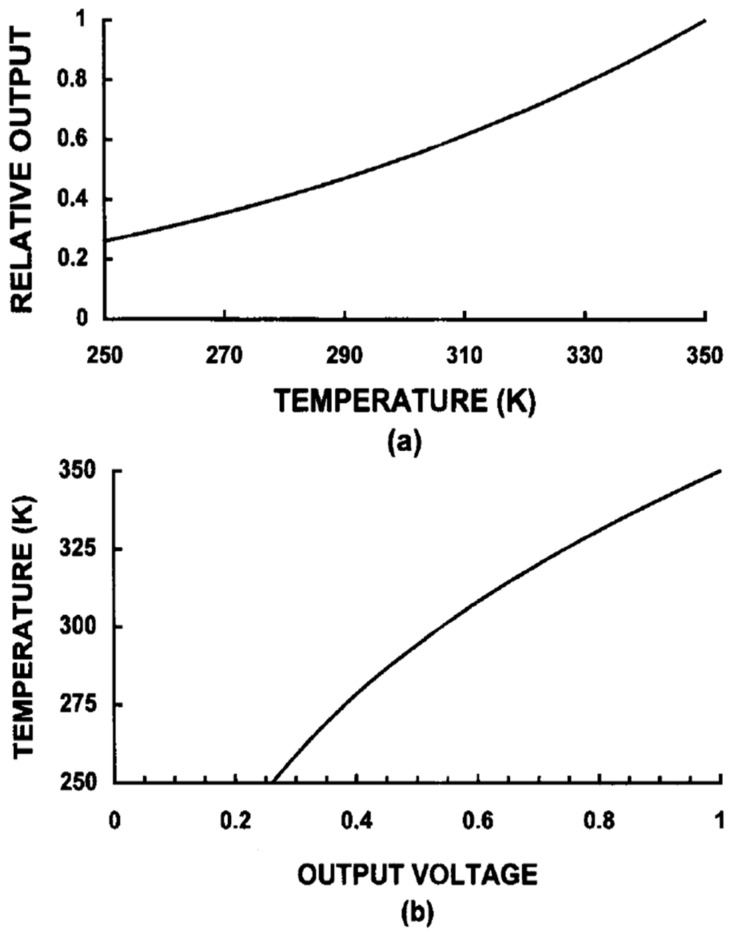
Temperature calibration: (**a**) Voltage output as a function of temperature obtained in a laboratory. (**b**) The temperature as a function of voltage. This calibration appears in a look-up table. Although the output is shown as an analog voltage, most systems operate in the digital domain [38].

Calibration is used to calculate a temperature proportional output signal (IR or thermal image) from the measurement signal (raw image) taking into account all technical and physical properties of the IR camera [39]. The calibration is executed on a number of blackbody measurements at known temperatures, radiance levels, emissivities, and distances. This creates a table of values based on the A/D counts from the temperature/radiance measurements. Whereby, a series of calibration curves are created for each condition and stored in the camera system’s memory as a series of numeric curve-fit tables that relate radiance values to blackbody temperatures. When the system makes a measurement, it takes the digital value of the signal at a given moment, it fits it into the appropriate calibration table, and it calculates temperature [37]. Examples of calibration curves are shown in Figure 2 [38].

Moreover, it is worth noticing that thermal imaging technique is a radiometric measurement method. Radiometry is the measurement of radiant electromagnetic energy, associated with the IR spectrum. Thermal IR imaging is not based on the direct measurement of the temperatures, since thermal IR devices reveal the thermal energy of the radiations. The camera receives radiation from the target object, plus radiation from its surroundings that has been reflected onto the object’s surface. Both of these radiation components become attenuated when they pass through the atmosphere. Therefore, an overriding issue is matching the detector’s response curve to what is called an atmospheric window, i.e., the range of IR wavelengths that pass through the atmosphere with little attenuation.

Essentially, there are two of these windows, one in the [2–5.6] μm range, the short/medium wavelength (SW/MW) IR band, and one in the [8,9,10,11,12,13,14,15] μm range, the long-wavelength (LW) IR band. There are many detector materials and cameras with response curves that meet these criteria [37]. 

Since the atmosphere absorbs part of the radiation, it will also radiate some itself (Kirchhoff’s law). The total radiation power received by the camera can be expressed as Equation (2):

(2) $$W_{\mathrm{tot}}=\varepsilon \cdot \tau \cdot W_{\mathrm{obj}}+\left(1-\varepsilon \right)\cdot \tau \cdot W_{\mathrm{amb}}+\left(1-\tau \right)\cdot W_{\mathrm{atm}}$$

where ε is the object emissivity, τ is the transmission through the atmosphere, W_amb_ is the emitted energy from the object surroundings, and W_atm_ is the emitted energy from the atmosphere.

Thus, it is possible to distinguish three terms in Equation (2):

1. *Emission* from the object, i.e., $\varepsilon \cdot \tau \cdot W_{\mathrm{obj}}$:
2. *Reflected emission from ambient source*, i.e., $\left(1-\varepsilon \right)\cdot \tau \cdot W_{\mathrm{amb}}$, where (1 − ε) is the reflectance of the object (it is assumed that the temperature is the same for all emitting surfaces within the half sphere seen from a point on the object’s surface);
3. *Emission from the atmosphere*, i.e., $\left(1-\tau \right)\cdot W_{\mathrm{atm}}$, where (1 − τ) is the emissivity of the atmosphere.

To arrive at the correct target object temperature, the IR camera software requires inputs for the emissivity of the object, atmospheric attenuation and temperature, and temperature of the ambient surroundings. Depending on circumstances, these factors may be measured, assumed, or found from look-up tables. In order to determine the values of the temperature measured by each detectors, Planck’s law is applied (Equation (3)):

(3) Wtot(λ,T)=ε(λ)·2πhc2λ5(ehcλkT−1)×10−6 [W/m2, μm]

where $W_{b}$ is the blackbody spectral radiant emission at wavelength λ, c = 3 × 10^8^ m/s is the velocity of light, h = 6.6 × 10^−34^ J·s is Plank’s constant, k = 1.4 × 10^−23^ J/K is Boltzmann’s constant , T is the absolute temperature of the blackbody and λ is the wavelength. Equation (3) shows the spectral distribution of the object IR radiation. Integrating Equation (3) over an appropriate spectral band, it is possible to obtain the total emittance of the object (Equation (4)):

(4) Wtot(T)=∫λ1λ2ε(λ)·2πhc2λ5(ehcλkT−1)×10−6dλ [W/m2]

Equation (4) is usually integrated numerically or found by using lookup tables.

## 3. Applications in Psychophysiology

Thermal signatures of a variety of autonomic parameters have been identified. In particular, it has been demonstrated that, through bioheat transfer models, it is possible to estimate at a distance physiological parameters, such as the cardiac pulse, the breathing rate, the cutaneous blood perfusion rate, the sudomotor response, and psychophysiological responses, as the stress response. This section reviews the methods and the results for computational physiology and psychophysiology based on thermal IR imaging. A general introduction on the measurement procedures for thermal imaging on humans is described. 

### 3.1. General Procedures for Thermal Imaging on Human Body

Thermal IR imaging permits the estimation of the superficial thermal pattern of the object of measurement. When the object of measurement is the human body some consideration has to be taken into account. In 2015, Fernàndes-Cuevas et al. summarized all the factors influencing the use of IR imaging on humans [40]. The most important factors are listed below:

- The usage of vasomotor substances (i.e., coffee, tea, alchool, drugs, tobacco) has to be avoided by the subjects the day of the experimental session [41]. The effects of the intake of these substances would influence the cutaneous thermal pattern.
- The region of interset for the measurement has to be depilated at least 4 h before the examination and the usage of moisturizing cream, make up or nail polish (in case of measurement on hands or feet) has to be avoided [41].
- When executing a thermal imaging measurement, it is mandatory to control the tempearture and humidity of the experimetal room. International Academy of Thermology (IACT) guidelines [41] suggest a temperature range of 18–23 °C and a controlled humidity range. A humidity range between 40% and 70% is reported in [40]. It is adviceble to execute the measurement in a large room (minimal room size is 2 × 3 m [40]) with no direct ventilation on the subject and no direct sunlight (no windows or with curtains or blinds).
- The distance between the subject and the camera should be enough to fill the viewable image area as to maintain adequate spatial resolution and interpretation accuracy, and the camera has to be as much as possible orthogonal to the plane of the region of investigation, to maximize the flux of thermal energy revealed by the camera [41].
- Moreover, it is necessary an acclimatization phase of the subject within the 15 min before the experimental session [41]. This phase is usefull both for thermal acclimatization and equilibrium with the experimetal room and stabilization of the emotianal status of the subjects.

It is recommended to refer to IACT guidelines [41] and to [40] for further details.

### 3.2. Computational Physiology

#### 3.2.1. Cardiac Pulse

Thermal IR imaging is able to estimate the cardiac pulse wave at a distance through the modelling of the pulsatile propagation of blood in the circulatory system [10,42,43,44,45]. Indeed, when the heart contracts during the ventricular systole, it generates a pressure wave, which propagates through the arterial system. The arterial pulse reflects the heart activity thus providing a measure of cardiac inter-beat intervals, heart rate, and its variability [44]. Garbey and colleagues [10] introduced a novel method for the cardiac pulse estimation with thermography, based on the hypothesis that the temperature modulation due to pulsating blood flow produces the strongest variation on the temperature signal of a superficial vessel. This method is based on the information contained in the thermal signal emitted from major superficial vessels and recorded through a highly sensitive thermal imaging system. The proposed model describes the heat diffusion process on the cutaneous layer originating from the core tissue and a major superficial blood vessel. Noise effect coming from environment and instability in blood flow were taken into account in the model. Their simulation demonstrated that the skin temperature waveform is directly analogous to the pulse waveform, except for its smoothed, shifted, and noisy shape caused by the diffusion process. To compute the heart modulation (pulse) frequency, a line-based region along the vessel was considered. Then, the authors applied Fast Fourier Transform (FFT) to individual points along this line of interest, to capitalize on the pulse’s thermal propagation effect. Finally, they use an adaptive estimation function on the average FFT outcome to quantify the pulse. The experiments were conducted on 34 subjects, comparing pulse computed from thermal signal analysis method with ground-truth measurement, given by a standard contact sensor (piezoelectric device). The performance of the method ranged from 88.52% to 90.33% depending on the clarity of the vessel’s thermal signal. Sun et al. [42] applied the same method but working at 90 degrees across the direction of the target vessel. The overall accuracy of the mean pulse measurement using the new method improved to 92.1% with respect to Garbey method. The abovementioned methods were then improved by Bourlai et al. [43], through the application of these methodologies on an automatic tracked ROI and the introduction of noise reduction through a two-stage algorithm that discards problematic frames as a result of bad tracking. The new method was tested on 12 subjects and reduced the instantaneous measurement error from 10.5 to 7.8%, while it improved mean accuracy from 88.6 to 95.3%. 

More recently, Farag et al. [44,45] introduced an automatic method to determine arterial pulse waveforms through the use of thermal imaging. This method was based on the hypothesis of the quasiperiodic thermal pattern on the skin due to the arterial pulse to automatically detect the areas surrounding superficial arteries. Multiscale decomposition models, such as wavelet decomposition, were applied to each thermal image to extract those scales containing most of the arterial pulse information. The influence of irrelevant noise was thus minimized and the arterial waveform recovery was more accurate. The coarser scales were used to track the Region of Interest (ROI) while the finer scales were used to compute the arterial pulse through the periodicity detection (PD) algorithm: a Region of Measurement (ROM) was chosen within each ROI and different ROM configurations were tested (size, orientation, scale, and location); for each tested ROM, continuous wavelet analysis was run to remove high frequency noise and to extract arterial pulses structures; maxima were calculated from the resulting waveform which in turn correspond to the systolic peaks (used to compute heart rate, beat to beat, and heart rate variability). The PD algorithm identified the optimal ROM in terms of the periodicity of the waveform and of its relevance to the true arterial pulse propagation. Validation of the method on 8 subjects showed perfect matching with oximeter data [45].

#### 3.2.2. Breathing Rate

Breathing function consists of inspiration and expiration cycles during which heat exchanges occur between airflows and nostrils. These exchanges generate a periodic or quasiperiodic thermal signal in the proximity of the nostrils that oscillates between high (expiration) and low (inspiration) values. This phenomenon can be captured by thermal imaging system at a distance, achieving an accuracy of 96.43% [9].

**Figure 3:**
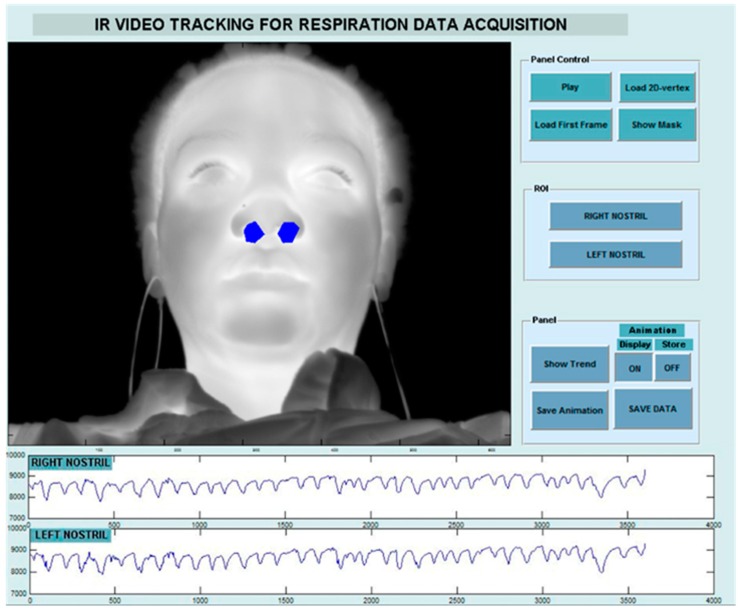
Thermal signal extracted from right and left nostrils regions [48].

In classical respiratory studies, a thermistor is usually positioned near the nostrils to capture this phenomenon and produce a representative breath signal [46]. Thermal imaging behaves therefore as a virtual thermistor, since it captures the same process, but at a distance. The estimation of breathing rate through thermal imaging is very accurate as proved by comparison with respiratory ground-truth signals acquired with conventional sensors [47,48]. Murthy et al. [47] found a high degree of chance corrected agreement (k = 0.92) between the airflow monitored through thermal imaging and oro-nasal thermistors. Correlation coefficients between the thermally and mechanically (LifeShirt technology; see [48]) recorded breath rate signals resulted maximized over a sample of 25 subjects, under shallow, normal, and forced ventilations [48]. Lewis et al. [48] showed also the possibility of estimating the relative tidal volume from thermal imaging (Figure 3). The correlation coefficient between the thermal and mechanical recordings over the same sample was 0.90. 

Statistical methods have also been proposed to compute the contactless breathing signature. The algorithm used by Murthy et al. [49] was based on the method of moments and Jeffrey’s divergence measure. This method has been tested on 10 subjects leading to a mean accuracy of 92% compared with the respiratory belt data at the thorax. Multiresolution analysis has been used as well [19,50]. Fei and Pavlidis [51] applied wavelet analysis in order to extract the breathing content from the mean temperature of the nostrils. They found a high degree of agreement between the thermally recovered breathing waveform and the corresponding thermistor over a sample of 20 subjects. In the work of Chekmenev et al. [50], the nasal region was tracked over time and for each frame the ROI was decomposed and averaged at three different scales. Wavelet transform was then applied to the resulting signal. The scale that contains most of the breathing information was extracted and used to compute the breathing rate. This approach has been tested on four subjects and the results perfectly matched the piezoelectric measure device signals.

Thermal IR imaging has been also used to recover breath-related thermal variations from nasal, ribcage, and abdomen regions of interest in children, both healthy and with respiratory pathology. Goldman [52] proved that thermal IR imaging reliably acquires time-aligned nasal airflow and thoraco-abdominal motion without relying on attached sensor performance and detects asynchronous breathing in paediatric patients. Fei and colleagues [53] introduced a novel methodology to monitor sleep apnea through thermal imaging. The nostril region was segmented and tracked over time via a network of cooperating probabilistic trackers. Then, wavelet decomposition was applied on the average thermal signal of the nostril region, carrying the breathing information. The experimental set included 22 subjects (12 men and 10 women). The sleep-disordered incidents were detected by both thermal and standard polysomnographic methodologies. A high accuracy level was achieved, thus confirming the validity of the proposed approach for nonobtrusive clinical monitoring of sleep disorders [53].

**Figure 4:**
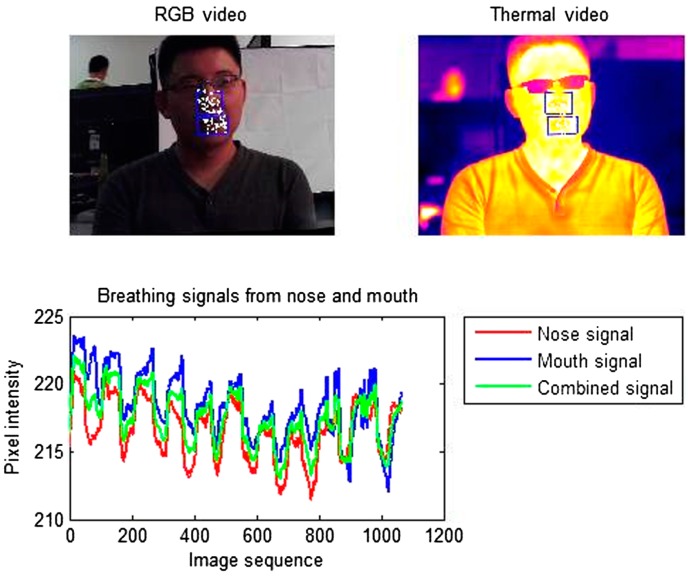
Screenshot of the breathing signature processing interface for visible and thermal dual-mode imaging system [54].

More recently, Hu et al. [54] used a dual system based on both visible and thermal imaging to monitor the breathing function. Visible images were used for face, nose and mouth detection through a cascade classifier based on Viola-Jones algorithm and for the tracking of these regions over time. ROIs coordinates were then used to extract the breathing signal on thermal video. An example of the procedure is shown in Figure 4. In terms of breathing rate estimation, the method achieved a correlation of 0.971 with a reference method and the Bland-Altman plot with 95% limits of agreement ranged from −2.998 to 2.391.

#### 3.2.3. Cutaneous Blood Perfusion

**Figure 5:**
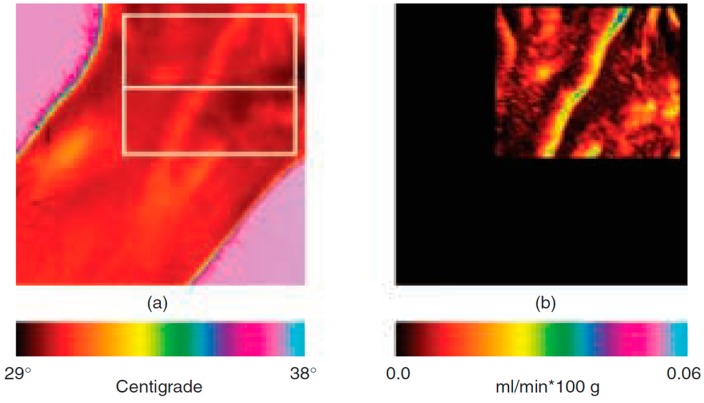
(**a**) Raw thermal image of a human wrist. (**b**) The corresponding blood flow rate visualization image. The color bars at the bottom index colors to values from the minimum to the maximum of the respective ranges [56].

Bioheat transfer models allow the estimation of the cutaneous perfusion from high resolution IR image series [55,56]. Pavlidis and Levine [56] suggested to use cutaneous perfusion rate changes in the periorbital region as a performing channel for a new generation of deception detection systems, based on the flight-fight response of the inquired subject to sensitive questions. An application of the method on the wrist is reported in Figure 5. 

The models adopted are derived from previous works of Fujimasa et al. [57], Pavlidis and Levine [56], and Merla and colleagues [55]. According to these models, cutaneous temperature change over a short time is mainly due to the heat gain/loss via convection attributable to blood flow of subcutaneous blood vessels and the heat conducted by subcutaneous tissue. The models showed that the blood flow rate and the cutaneous blood flow depend mostly on the time-derivative of the cutaneous temperature and on the difference between the temperatures of the cutaneous layers and the inner tissues [55]. It has been demonstrated that it is therefore possible to transform raw thermal image series in cutaneous blood flow image series. The method has been validated by comparison with laser Doppler imagery. Merla and colleagues showed that, in 20 healthy subjects, cutaneous blood flow values, simultaneously computed by thermal IR imagery and measured by laser Doppler imaging, linearly correlate (R = 0.85, Pearson Product Moment Correlation) [55]. The method has been applied in psychophysiology for deception detection [56] and emotion assessment [2].

Furthermore, in 2009, Gorbach et al. compared IR imaging data with full-field laser perfusion imager (FLPI) to assess vascular responses of the human hand to inspiratory gasp and hand cooling [58]. The highest spatial correlation was found between the mean derivative IR image and the mean raw FLPI image for the baseline condition. After cooling, a temperature increase of ~0.5 °C was observed in thenar and hypotenar areas; in the same areas, an increase of perfusion was observed through FLPI. The combination of both IR imager and FLPI was considered an ideal approach to investigating the dynamics of thermal and perfusion heterogeneity in human skin. 

#### 3.2.4. Sudomotor Response

Electrodermal responses represent one of the most widely employed psychophysiological measures of autonomic nervous system activity. The Skin Conductance Response (SCR) and related measures, like galvanic skin response (GSR), have been shown to correlate with the number of active sweat glands, which activation can be easily visualized through facial thermal IR imaging by the appearance of cold dots over the face. Together with the palm area, strong sweat gland activation is shown in the maxillary, perioral, and nose tip regions.

Multiresolution analysis of the temperature signals reveals tonic (baseline and/or general) and phasic (event-related) components strongly correlated with GSR sympathetic constituents [12,13,16,59]. For example, Pavlidis et al. [13] showed very high correlation coefficients between the GSR and the thermal measurement on the finger (r_MIN_ = 0.968) and on the perinasal region (r_MIN_ = 0.943).

Moreover, it has been demonstrated that the maxillary area contains information about the sympathetic response almost as much as the GSR channel [12]. As a support of this founding, several studies showed that the identification of active eccrine sweat glands by thermal imaging may have utility as a psychophysiological measure of sudomotor activity and may substitute the GSR signals when a contact method is either unavailable or undesirable [2,5,12,16,60]. 

**Figure 6:**
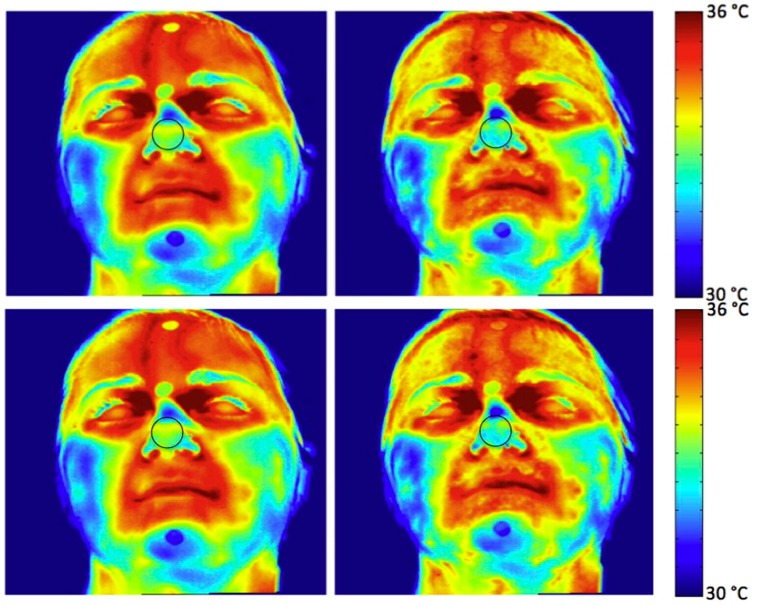
Facial thermal changes in a representative subject. On the left: the average temperature before the acoustic stimulation; on the right: soon after the acoustic stimulation. A general temperature drop can be observed over the whole face, with the onset of sudomotor response as highlighted by the dotted pattern of the temperature associated with the emotional sweating response. In particular, while the cheeks do not change their average temperature values and pattern, nose tip, perioral, maxillary and forehead regions clearly present a temperature decrease due to the appearance of colder dotted spots. The temperature decrease is particularly appreciable on the nose tip, where blue areas can be easily spotted. The circles over the face are paper markers put on to facilitate the tracking of the region of interest along the procedure. The black-contour circular region on the nose tip is the region of interest from which the temperature data have been extracted [61].

Recently, thermal IR imaging has been applied, together with standard GSR, to examine fear conditioning in posttraumatic stress disorder (PTSD) [61]. The authors studied fear processing in PTSD patients with mild symptoms and in individuals who did not develop symptoms, through the study of fear-conditioned response (Figure 6). The authors found that the analysis of facial thermal response during the conditioning paradigm performs like GSR to detect sympathetic responses associated with the disease.

### 3.3. Computational Psychophysiology

#### 3.3.1. Stress Response

IR thermal imaging is a suitable technique in the stress research field, because of its non-invasiveness. In 2007, Or et al. conducted a study about occupational ergonomics on professional drivers, assessing mental workload using thermal IR imaging. The authors studied the thermal response on subjects executing simulator driving tasks both in the city and on the highway while cognitively engaged with a Mental Loading Task (MLT). Significant differences were observed in the nose temperature across all conditions, compared to the baseline session (pre-driving). The MLT seemed to have an influence on the temperature decrease of the nose, during the simulated city drive. No significant effects were observed on the forehead [62].

In 2012, Pavlidis and colleagues [13] tried to assess the stress level by measuring transient perspiratory responses on the perinasal area using thermal IR imaging. These responses proved to be a good indicator of stress response, because sympathetically driven. The authors applied this approach in the context of surgical training and, more recently, on drivers [63]. The effects of cognitive, emotional, sensorimotor, and mixed stressors was studied on 59 drivers in a simulation experiment. Perinasal perspiration, revealed by thermal imaging, together with the measure of steering angle and the range of lane departures on left and right side of the road showed a more dangerous driving condition in case of sensorimotor and mixed stressors with respect to the baseline condition. A safer driving was adopted in case of emotional and cognitive stressors. 

Besides, concerning the human-computer interaction field, Puri et al. [64] and Zhu et al. [65] used a Stroop task to elicit signs of frustration. Based on frontal regions, they observed that, compared with the resting state, stress increased blood volume into supraorbital vessels. Kang et al. [66] used thermal IR imaging to assess affective training times by monitoring the cognitive load through facial temperature changes. Learning proficiency patterns were based on an alpha-numeric task. Significant correlations, ranging from −0.88 to 0.96, were found between the nose tip temperature and the response time, accuracy, and the Modified Cooper Harper Scale ratings. Through this work, the authors demonstrated that thermal IR imaging is a sensitive tool to assess learning and workload. Engert et al. [15] explored the reliability of thermal IR imaging in the classical setting of human stress research. Thermal responses were compared to gold standard stress markers (heart rate, heart rate variability, finger temperature, alpha-amylase, and cortisol) in healthy subjects participating in two standard and well-established laboratory stress tests: the cold pressor test [67] and the trier social stress test [68]. Both tests showed evidence of thermal responses of several regions of the face. The authors found a weak correlation between the thermal responses and the established stress markers but, on the other hand, the thermal imprints correlated with stress-induced mood changes. In contrast, the established stress markers did not correlate with stress-induced mood changes. These results suggested that thermal IR imaging is an efficient technique for the estimation of sympathetic activity in the stress research field.

#### 3.3.2. Social Interactions

In the prospective of studying the human emotional and affective state, it’s very interesting to investigate what happens when two or more persons are interacting. Nowadays, many studies have been conducted on social interaction using thermal imaging. Indeed, thanks to its non-invasiveness, it is considered one of the most suitable method in this research field. Aureli et al. [69] studied the interaction between mother and her own infant (3–4 month old babies) during the Still-Face Paradigm [70]. Infants interacted with their mothers during a four phase experiment: (i) normal interaction episode, in which the mother freely interacted with her infant; (ii) “still-face” episode, in which the mother stopped playing with her infant and became unresponsive and with a neutral facial expression; (iii) reunion episode, in which the mother resumed the interaction; (iv) toy-play episode, in which the mother added a toy during the interaction. From the behavioural analysis, the authors found that infants recognized the interruption of the interaction during the still-face episode and increased the engagement with the environment, decreasing their communicative engagement. Concerning the thermal data analysis, they observed an increase in temperature on the nose tip and on the forehead comparing the still face episode to the toy-play condition. Thermal data were, thus, consistent with behavioural data, since they reflected a stronger activation of the parasympathetic with respect to the sympathetic system. 

**Figure 7:**
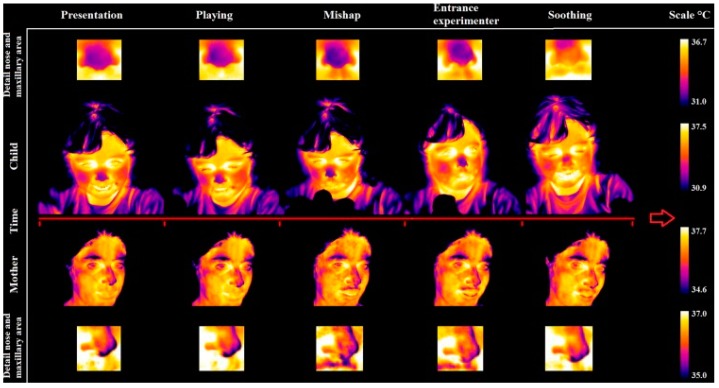
Facial thermal imprints of one of the mother–child dyads [71].

**Figure 8:**
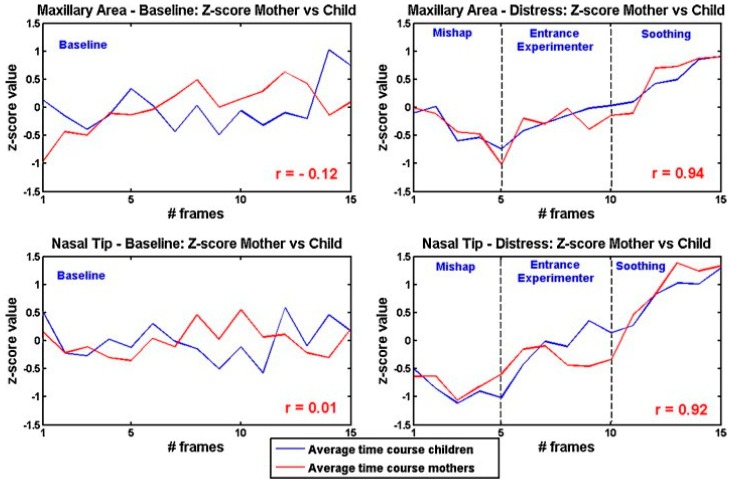
Graphical representation of group temperature variations of the nasal tip and maxillary area during the experimental phases as well as during the neutral baseline period [71].

Ebisch et al. [71] focused on the interaction between mother and her own child (pre-scholar age child). They studied whether maternal empathy is concomitant with synchrony in autonomic responses between mother and child. They analysed simultaneously the facial thermal response of mother and her own child, while the last was involved in a distressing situation. The mother observed her own child through a one-way mirror. The results showed evidence for a direct affective sharing, involving autonomic responses. An example of the thermal pattern recorded on a mother-child dyad is showed in Figure 7. In Figure 8, a graphical representation of temperature variation of mother and child groups during baseline and experimental phases is reported.

The study was then extended on an additional group of females, who were not mothers of the child they were looking at (other women) [72]. The results showed that mother-child dyads have a faster empathic responses to the child’s emotional state than other women-child dyads. 

Güney et al. [73] reported a case study, examining the intra- and inter-personal emotion regulation of a patient with somatic symptom disorders (SSDs) while interacting with his romantic partner, in comparison with a healthy control couple. The couples experienced a baseline, anger and relaxation tasks experiment. Measuring participants’ facial temperature, heart rate and GSR, they found significant differences with respect to healthy controls couple. In particular, all participants, except patient's partner, experienced an increase in temperature from baseline to relaxation. This was probably due to a complementary down-regulation of physiology in her interaction with the patient. The patient showed also a higher mean value of GSR and heart rate then his partner throughout all the experimental phases, reflecting a higher autonomic activity regulation.

A virtual social interaction has been the matter of study of Paolini et al. [74]. They assessed the autonomic response of the subjects during ostracism experience in the context of an online ball tossing game (i.e., Cyberball). This autonomic response is stronger when individuals are ostracized by teammate (vs. outgroup) members. Similar pattern of temperature variations emerge when individuals observe an ostracism episode involving ingroup members.

Thermal IR imaging has been recently used also in interaction between humans and “social robots”. A first attempt has been made in the Robot AVatar thermal-Enhanced (RAVE) prototype project: a robot engage babies’ interest and identify babies’ emotional arousal signatures of being “ready to learn”, classifying the facial thermal responses of the babies. In turn, the robot will make available to the baby socially interactive and spontaneous language samples assigned to them with virtual humans [20,21].

## 4. Discussion

Thermal IR imaging has been proved to be a reliable method for ubiquitous and automated monitoring of psychophysiological activity. It provides a powerful and ecological tool for computational physiology. The reliability and validity of this technique were proven by comparing data simultaneously recorded by thermal imaging and gold standard methods, as piezoelectric pulse meter for pulse monitoring, piezoelectric thorax stripe or nasal thermistors for breathing monitoring, skin conductance, or galvanic skin response (GSR). Concerning the latter, several studies have demonstrated that funcional Infrared Imaging (fIRI) and GSR have a similar detection power [12,13,15,59,61]. These results rely on the impressive advancement of the technology for thermal IR imaging. Modern devices ensure a high spatial resolution (up to 1280 × 1024 pixels with up to a few milliradians in the field of view), high temporal resolution (full-frame frequency rate up to 150 Hz), and NEDT values up to 20 mK at 30 °C in the spectral range [3–5] µm. The commercial availability of 640 × 480 focal plane array of uncooled and stabilized sensors (spectral range 7.5 ÷ 13.0 µm; full-frame frequency rate around 30 Hz; thermal sensitivity around 30 mK at 30 °C) allows this technology to be integrated into automated systems for remote and automatic monitoring of physiological activity. Real-time processing of thermal IR imaging data and data classification for psychophysiological applications is possible as the computational demand is not larger than that required for 640 × 480 pixels visible-band imaging data [5,18,75]. Thermal IR imaging has been indicated as a powerful tool to create, given the use of proper classification algorithms, an atlas of the thermal expression of psychophysiological responses [76,77]. This would be based on the characterization of the thermal signal in facial regions of autonomic valence (nose or nose tip, perioral or maxillary areas, periorbital and supraorbital areas associated with the activity of the periocular and corrugator muscle and forehead), to monitor the modulation of the autonomic activity. Several studies have already shown the possibility of using thermal IR imaging in psychophysiology (see [5,78] for reviews). These studies cover a number of fields, including developmental psychology and maternal empathy [71,72,79], social psychology [15,80], and up to lie detection [56,81,82]. 

However, several limitations exist for using thermal IR imaging in the real world. In order to avoid to misinterpret the thermal reactions of the body, attributing physiological valence to mere thermoregulatory or acclimatization processes, it is mandatory to take adequate countermeasures, concerning environmental conditions [5,40,41]. Besides, despite of the advantages offered by thermal IR imaging, it has to be taken into account that thermal signal development as a result of vascular change, perspiration, or muscular activity is rather slow with respect to other established techniques. Proper considerations should therefore be taken when monitoring thermal signatures of psychophysiological activity. 

Taking into account these limitations, there is the concrete possibility of monitoring, in a realistic environment, at a distance and, unobtrusively, several physiological parameters and affective states. This is, in particular, possible thanks to tracking algorithms applied on thermal IR videos. Several studies demonstrated the feasibility of applying tracking procedures on thermal images with high accuracy [18,19], in order to obtain a reliable and stable monitoring of the thermal pattern of one or more ROIs over time. 

This opens the way for remote monitoring of the physiological state of individuals without requiring their collaboration and without interfering with their usual activities, thus suggesting the possibility of adding information of psychophysiological valence to behavioural or other typologies of investigation. One still unexplored but intriguing aspect is the study of possible correlation between individual thermal signatures and psychometric indexes, in order to assess, for example, whether given personality traits lead to inter-individual differences in the facial thermal signature of autonomic activity or affective state or whether specific thermal expressions of specific personality or sociality traits exist. Of course, thermal IR imaging is not the first and unique attempt to explore these possibilities [83,84], but thermal IR imaging seems to be one of the most ecological ones in this perspective. As such, thermal IR imaging provides an extraordinary opportunity to add physiological information to psychometric features, toward more robust classification of the individual’s affective states, emotional responses, and profile. A major issue that needs to be addressed for the practical application of thermal IR imaging in support of psychometrics concerns the adequacy of the method for identifying specific emotional or affective state at individual level. A first attempt in this perspective has been done by Cruz-Albarràn and colleagues [85]. In a recent work, they used a top-down hierarchical classifier to detect human emotions (i.e., joy, disgust, anger, fear and sadness) on 25 subject through thermal IR imaging. They were able to classify the emotions with an accuracy level of 89.9%. Apart from this recent and preliminary study, there is still an open issue concerning the specificity of the technique for the identification of specific emotional states and their corresponding thermal signatures. A definitive answer to this question is currently not available, given the fact that it is still not very well assessed how specific it is the autonomic reaction to different emotional states and their combination.

Finally, it is worth noticing that the impressive spread of IR technology, together with the miniaturization of IR detectors, conducted the manufacturer houses to produce mobile thermal cameras. During the last years, indeed, FLIR (Wilsonville, OR, USA) and Opgal (Karmiel, Israel) were the first companies to commercialize mobile thermal devices designed to be integrated on mobile phones. Some of the features are reported in Table 1.

While FLIR One Pro^®^ and Opgal Therm-App^®^ TH are strictly designed for mobile applications for smartphone and tablets, FLIR Lepton^®^ is customizable. Thanks to its microscopic dimensions, it is fully embeddable on several devices, offering the possibility of both integrating into native mobile-devices or other electronics as an IR sensor or thermal imager.

## 5. Conclusions

The impressive spread of IR detectors technology together with the development of biological models for computational physiology and psychophysiology, based on thermal IR imaging, open the way for an innovative and up-to-date approach of thermography in the field of neurosciences. The development of miniaturized and mobile thermal cameras offer the concrete possibility of monitoring at a distance and, unobtrusively, several physiological parameters and affective states of everyday life experiences and will constitute definitely the core of the future research in this area.

## Figures and Tables

**Table 1:** Technical features of modern mobile thermal devices.

Feature	FLIR One Pro^®^	FLIR Lepton^®^ Radiometric	Opgal Therm-App^®^ TH
Size (w × h × d)	68 × 34 × 14 mm	11.8 × 12.7 × 7.2 mm	55 × 65 × 40 mm
Weight	36.5 g	0.9 g	123 g
FPA	160 × 120 pixels	80 × 60 pixels	384 × 288 pixels
NEDT	0.15 °C	<0.05 °C	<0.07 °C
Operating temperature range	0 °C to +35 °C	−10 °C to +80 °C	−10 °C to +50 °C

## Appendix A. Measurement Properties and Specification of IR Thermal Camera

A number of factors qualifies the performances of a thermal imaging system. These are:

*Repeatability*. A particular camera, under the same environmental conditions, will have a statistical repeatability of measurement, typically close to the NETD value, which is the amount of incident signal temperature that would be needed to match the internal noise of the detector such that the signal-to-noise ratio is equal to one. Typical value of this parameter is below 20–30 mK @ 30 °C for cooled detectors and 30–50 mK @ 30 °C for good quality microbolometers.

*Accuracy.* Most thermal cameras have a specified accuracy of ±2 °C or ±2% of reading (sometimes also ±1 °C or ±1%). This means that the camera, under any environmental condition (within specification), at any time, will give a reading within the accuracy specification.

*Stability.* The thermal stability of a sensor is defined as the percentage possible error in the measurement per unit (K or °C). This kind of error is caused by several factors, like the physical expansion of embedded components or the effect of thermal fluctuation on the performance of electronic components of the detector. These effects causes the thermal drift error, i.e., a slight deviation of measurement result per °C.

*Optics and focal length.* Thermal cameras lenses are not composed of simple regular glass, as glass reflect thermal radiation rather than allowing it to pass through the lenses. Commonly used materials for thermal lenses are germanium (Ge), chalcogenide glass, zinc selenide (ZnSe) and zinc sulfide (ZnS). They are characterized by good transmission for wavelengths in the LW (long-wavelength) IR range ((8–15) µm). Lenses are commonly identified by their focal length *f*, defined as the distance from the front of the lens to the point at the back where it focuses the energy of the object of measure. It is expressed in millimeters.

*Field of View (FOV)**. FOV* is the subtended angle (expressed in angular degrees or radians per side if rectangular, and angular degrees or radians if circular) over which the optical system will integrate all incoming radiant energy. It is a parameter depending on the camera lens and on the focal plane array dimension. It is related to the focal length, as shown in Equation (A1):

(A1) $$\mathrm{FOV}=\tan^{-1}\frac{d}{2f}$$

where d = Focal Plane Array diagonal (mm), f = focal length (mm), FOV = field of view (degrees). According to (1), as the focal length increases, the field of view for that lens will be narrower and viceversa.

*Instantaneous Field of View (IFOV).* The IFOV corresponds to the angular projection of the detector element on the target [86]. It defines the spatial resolution, i.e., the size of smallest object that can be viewed or resolved at a specific distance from the camera.
